# Inhibiting PTEN Protects Hippocampal Neurons against Stretch Injury by Decreasing Membrane Translocation of AMPA Receptor GluR2 Subunit

**DOI:** 10.1371/journal.pone.0065431

**Published:** 2013-06-17

**Authors:** Yuan Liu, Li Wang, Zai-yun Long, Ya-min Wu, Qi Wan, Jian-xin Jiang, Zheng-guo Wang

**Affiliations:** 1 Department of Research Institute of Surgery, Daping Hospital, the Third Military Medical University, State Key Laboratory of Trauma, Burns and Combined Injury, Chongqing, China; 2 Department of Physiology and Cell Biology, University of Nevada School of Medicine, Reno, Nevada, United States of America; Hertie Institute for Clinical Brain Research, University of Tuebingen, Germany

## Abstract

The AMPA type of glutamate receptors (AMPARs)-mediated excitotoxicity is involved in the secondary neuronal death following traumatic brain injury (TBI). But the underlying cellular and molecular mechanisms remain unclear. In this study, the role of phosphatase and tensin homolog deleted on chromosome 10 (PTEN) in GluR2-lacking AMPARs mediated neuronal death was investigated through an in vitro stretch injury model of neurons. It was indicated that both the mRNA and protein levels of PTEN were increased in cultured hippocampal neurons after stretch injury, which was associated with the decreasing expression of GluR2 subunits on the surface of neuronal membrane. Inhibition of PTEN activity by its inhibitor can promote the survival of neurons through preventing reduction of GluR2 on membrane. Moreover, the effect of inhibiting GluR2-lacking AMPARs was similar to PTEN suppression-mediated neuroprotective effect in stretch injury-induced neuronal death. Further evidence identified that the total GluR2 protein of neurons was not changed in all groups. So inhibition of PTEN or blockage of GluR2-lacking AMPARs may attenuate the death of hippocampal neurons post injury through decreasing the translocation of GluR2 subunit on the membrane effectively.

## Introduction

Traumatic brain injury (TBI) is one of the major cause of death and permanent disability in traumatic patients [1], [2]. Neuronal degeneration following TBI is believed to involve in primary mechanical injury and progressive secondary injury [1]. However, the underlying mechanism of secondary injury in TBI is not clear entirely. So far, alteration in excitatory amino and its receptor is regarded as a critical cause for the progressive neuronal death following TBI [3], [4].Glutamate is the most abundant excitatory neurotransmitter in the brain. Increasement of extracellular glutamate following brain injury will lead to over**-**stimulation the function of glutamate receptors, such as AMPA, NMDA receptor, that may result in secondary injury and causing the death of neuronal cells [5].

AMPA receptors (AMPARs) mediate fast synaptic transmission at excitatory synapses of neurons in the central nervous system(CNS) and are assemblies of GluR1**-**4 subunits, which are differentially expressed throughout the CNS [6].The GluR2 subunit governs a number of characteristics of AMPARs, among which AMPARs containing GluR2 subunit are impermeable to divalent cations and protect neurons against injury caused by influx of Ca^2+^ and Zn^2+^. AMPARs lacking GluR2 subunit are permeable to Ca^2+^ and Zn^2+^ [7].

Under physiological conditions, the neurons in hippocampus abundantly express GluR2**-**containing Ca^2+^-impermeable AMPARs. However, recent studies indicated that Ca^2+^-permeable GluR2-lacking AMPARs may play a crucial role in the excitotoxicity in TBI [8]. Although considerable evidence identified the alteration in AMPAR subunits composition and function after CNS injury, the regulation of GluR2 subunit trafficking in TBI remains unclear [5], [9], [10]. Thus, understanding the molecular mechanisms regulating AMPARs may provide the possibility of developing effective drugs for preventing traumatic neuronal death in nervous system.

The tumor suppressor PTEN (phosphatase and tensin homolog deleted on chromosome 10) is a lipid and protein phosphatase, which can regulate cell cycle, cell migration and growth. Recent studies have shown that suppressing PTEN protects ischemic neuronal death by enhancement of Akt activation and inhibition of NMDA receptor in *vitro* and in *vivo* [11]. Further studies indicated that downregulation of PTEN promote the survival of ischemic neurons via preservation of GABAAR on the surface of membrane [12]. These evidences suggest that PTEN play a crucial role in regulating expression of both excitatory and inhibitory ion channels receptors on neurons. In the present study, we examined changement of GluR2 and effect of PTEN suppression on AMPAR GluR2 subunit expression in cultured hippocampal neourons following traumatic injury in *vitro*, it was demonstrated that PTEN-induced reduction of GluR2 subunit of AMPARs on the surface of membrane contributes to neuronal death after injury.

## Materials and Methods

### Hippocampal neurons culture and stretch injury in vitro

Hippocampal neurons were prepared from Wistar rats at gestation day 18 [13]. Mechanically dissociated of hippocampal neurons were suspended in plating medium (Neurobasal medium (NB, Gibicol, USA), 2% B**-**27 supplement (Gibicol, USA), 0.5% FBS (Gibicol, USA), 0.5 µM L**-**glutamine (Sigma, USA) and transferred to poly**-**D**-**lysine**-**coated coverslips in 6**-**well plate with flexible collagen**-**coated silicone rubber membranes at the bottom of each well (Flexcell). After 3 d in vitro, three**-**quarters of the plating medium was removed and replaced with maintenance medium (NB medium, 2%B**-**27 supplement and 0.5 µM glutamine). Medium replacement was performed every 3–4 d and cells were ultilized at 12–15 d after culture. Mechanical stretch was applied to cultured neuronal cells *in vitro* using a modification strain unit as described [14]. A vacuum (25 kPa) was applied from the base of the plate for 2 seconds. The maximal percent elongation of the culture surface was 30% [15]. Cells cultured on the same type of plates without stretch were served as control.

The normal cultured neurons at the same time were regarded as control. For the sham+bpv/Nas group, the cultures were treated with 200 nmol/L bisperoxo (pyridine-2-carboxyl) oxovanadate(bpv,Alexis Corporation,Switzerland) or 20 µmol/L 1-naphthylacetyl spermine thihydrochloride (Naspm/Nas, Sigma, USA) in plating medium for 2 hours at 37°C in a 5% CO2 incubator without injury. For the stretch injury group, the neurons were subjected to stretch injury as described above without any treatment. For the stretch injury+bpv/Nas group, cells were given 200 nmol/L bpv or 20 µmol/L Nas in plating medium for 2 hours and then subjected to the stretch injury. In the injury+bpv+Nas group, cells were given 200 nmol/L bpv and 20 µmol/L Nas in plating medium for 2 hours and subjected to stretch injury. The cultured neurons in all different groups were further examined at corresponding time points.

### RT-PCR assay

Total RNA of hippocampal neurons in different groups was extracted using Trizol (Roche) at 6, 12, 24 hours after injury. RT was performed in a 20 µl reaction containing RNA 4 µl, OligodT (Takara) 1 µl, DEPC water 4 µl, at 65°C for 10 min and on ice for 5 min; moreover, added RNAase inhibitor 0.5 µl, 5×buffer 4 µl, 10 mM dNTP 2 µl, AMV (Takara) 1.5 µl and DEPC water 3 µl, at 42°C for 90 min in PCR machine. The 25 µl PCR reaction additionally contained the following components: 1 µl cDNA, 0.5 µl of each primer, Tag master mixture 12,5 µl (including dNTP mixture, tag plus DNA polymerase)and ddH2O 10.5 µl (Takara). The PCRs were conducted in a programmable thermocycler (Thermo,USA) using an initial denaturing temperature of 94°C for 2 min, 30 cycles of 94°C for 1 min, 56°C for 1 min, 72°C for 1 min. The sequences of primers were as follows [16]:

PTEN

Forward primer: 5′-AACCGATACTTCTCTCCAAAT-3′

Reverse primer: 5′-TTCATCAAAAGGTTCATTCTC- 3′

GAPDH

Forward primer: 5′-ACCACAGTCCATGCCATCAC-3′

Reverse primer: 5′-TCCACCACCCTGRRGCTGTA-3′

### Western blot analysis

Hippocampal neurons in culture were lysed and centrifuged at 14,000 rpm for 20 min at different time points. Protein concentration of the supernatants from extract was determined with BCA protein assay kit (Pierce Biotechnology Inc, USA) [17]. Equivalent amounts of protein samples were loaded on SDS**-**polyacrylamide gels. After electrophoresis, the proteins were transferred to PVDF membranes and the blots were subsequently probed with mouse anti**-**rat GluR2 monoclone antibody (1∶1000, Chemicon, USA), mouse anti**-**human PTEN monoclone antibody (1∶1000, Cell signaling, USA) and β**-**actin (1∶1000; Sigma**-**Aldrich) antibody overnight at 4°C,then the PVDF membranes were incubated with biotinylated secondary antibody for one hour in 5% non-fat milk in TBST. Immunoreactivity was detected by streptavidin alkaline phosphatase conjugate tertiary antibody. The optical density was quantified with the Image**-**Pro Plus 6.0 software. Separate experiments were conducted three times.

### PTEN expression vectors

An expression vector of PTEN was gifted from Qi Wan. Wild**-**type PTEN**-**GFP was transfected with lipofectamine 2000 (Invitrogen, USA) as described previously [11].

### Immunocytochemical staining

The expression of GluR2 on neurons membrane surface was labeled with mouse anti-rat GluR2antibody (Chemicon) and Alexa Fluor 488 (green fluorescence) secondary antibody (Invitrogen, Carlsbad, CA). Fluorescent-labeled receptors were imaged using a 63×objective mounted on a Leica (Germany) SP2 confocal microscope [18]. Images were analyzed using Image Element analysis software (Leica,Germany).Each image was a “flattened” into a single image using a maximum projection. For all experiments, we analyzed fluorescent signal in regions of interest by following method: Average fluorescence intensity per unit area was measured.The quantification of fluorescence staining was performed as described [19]. For individual experiments, all images in experiments were analyzed with identical acquisition parameters. For each experiment, control and treated cells from the same culture preparation were processed and imaged in parallel. Neurons were selected randomly under bright**-**field optics and fluorescent images of each neuron were acquired from a single plane for analysis in each experiment.

### Propidum iodide labeling

To determine the death of hippocampus neurons expressing PTEN at 24 h after transfection and stretch-injured hippocampus neurons 24 h post-injury, the neurons were cultured with plating medium containing 50 µg/ml propidum iodide (PI, Sigma, USA) for 10 min. Then the cultures were washed with D-Hanks three times and fixed with 4% paraformaldehyde for 20 minutes. The cells were labeled with mouse anti**-**rat neuronal nucleus antigen (Neon, Sigma, USA) antibody and goat anti**-**mouse Alexa Fluor 488 (green fluorescence) secondary antibody. The number of dead neurons was determined by calculating both PI and NeuN double labeling cells.

### Statistics

All population data were expressed as mean+SD. Statistical analysis of data was performed by a one-way analysis of variance (ANOVA). P<0.05 was considered to be statistically significant.

## Results

### 1. PTEN increased after stretch injury

**Figure 1:**
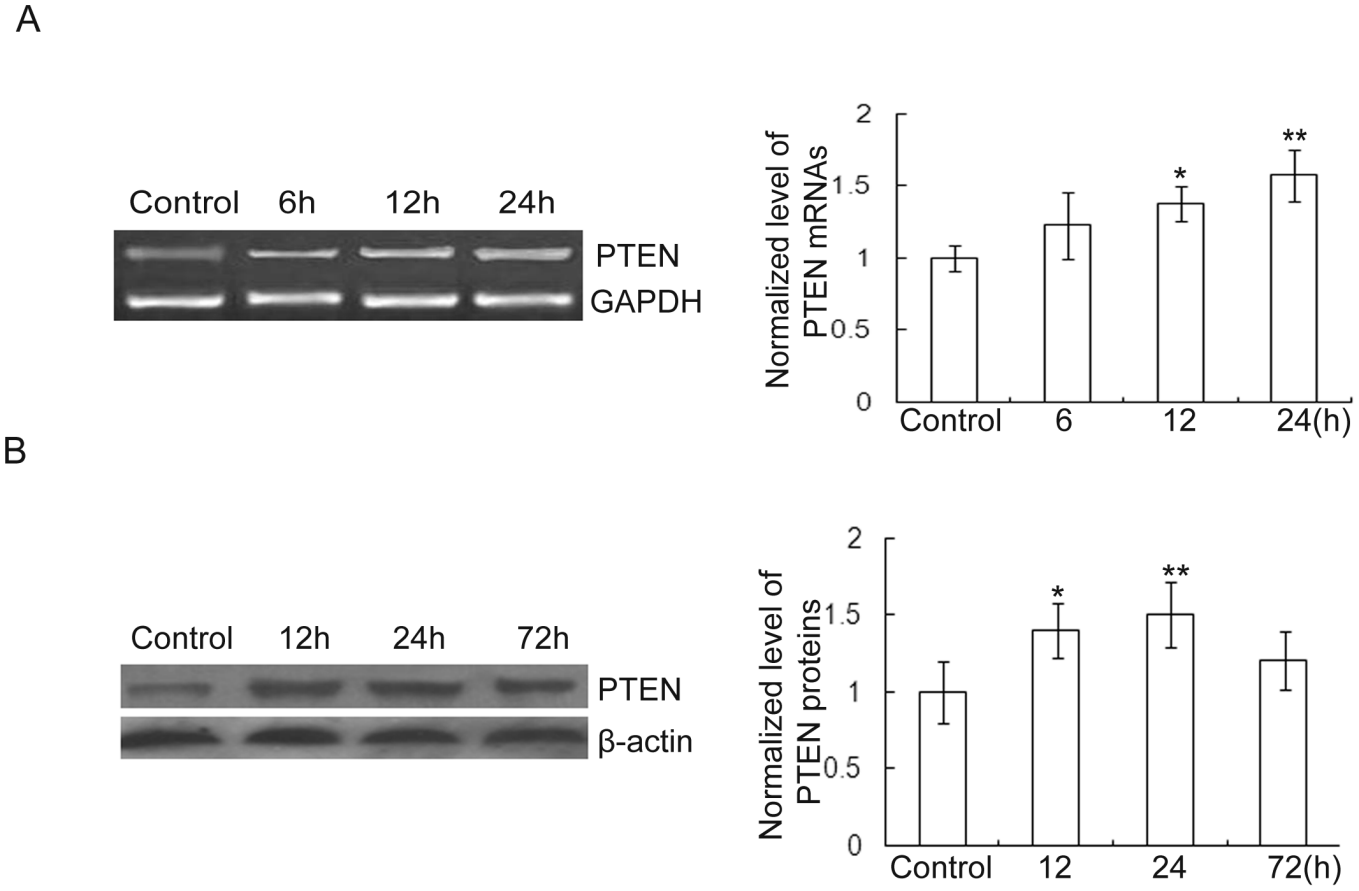
Expression of PTEN in cultured hippocampal neurons post stretch injury. A. Representative images of RT-PCR showing that the expression of PTEN mRNA increased post stretch injury in cultured rat hippocampal neurons (n = 3 for each group; Compared with normal group: **p<0.01, *p<0.05). B. Representative images of western blot showing that the expression of PTEN protein increased post stretch injury in cultured rat hippocampal neurons. (n = 3 for each group; Compared with normal group: **p<0.01, *p<0.05). β-actin was used as a loading control.

RT**-**PCR examination of injured neurons in culture indicated that PTEN mRNA begin to increase at 6 hours, evident at 12 and 24 hours post**-**injury(As illustrated in Fig. 1A). The expression of PTEN protein began to increase 12 hours after injury, significantly at 24 hours (as shown in Fig. 1B).

### 2. Stretch injury reduced GluR2 expression on neuronal membrane surface

**Figure 2:**
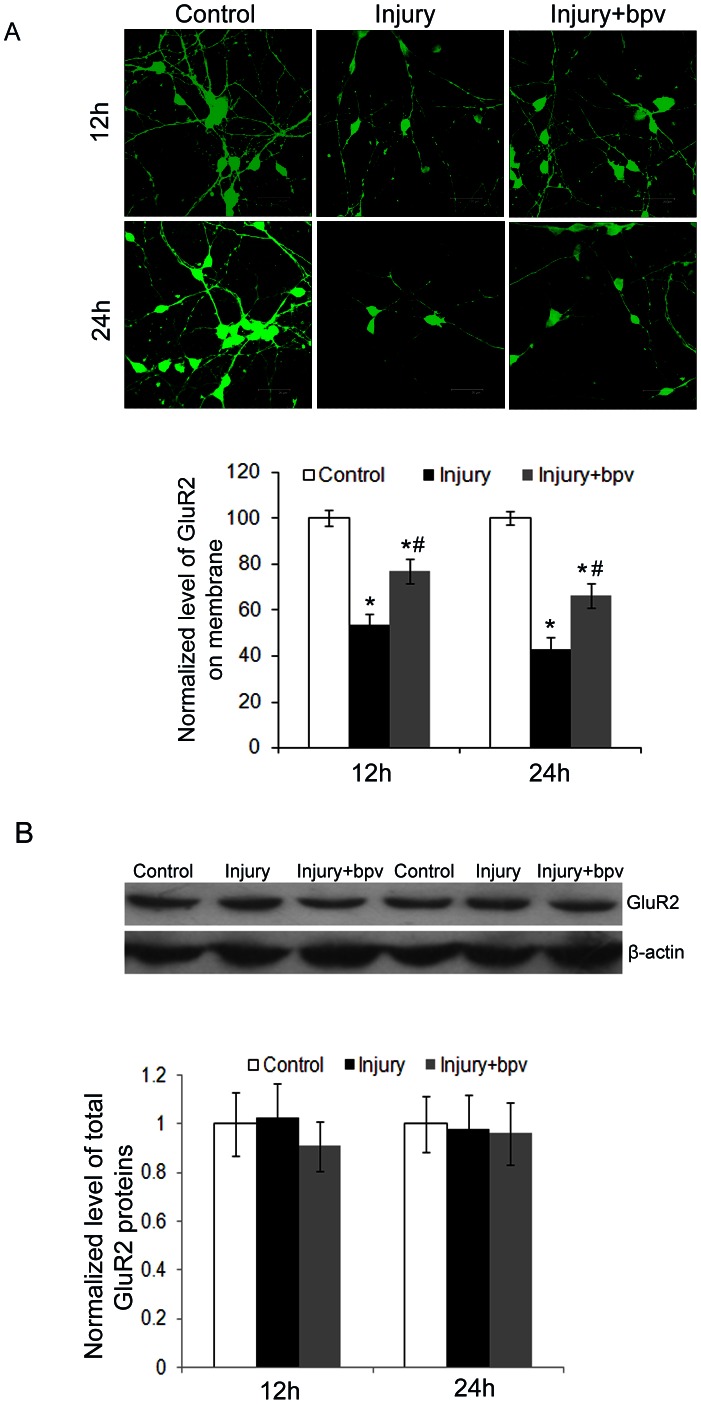
Changes of surface GluR2 subunit expression in cultured hippocampal neurons post stretch injury. A. Representative images showing that surface expression of membrane GluR2 subunits was reduced after injury, but inhibition of PTEN activity by bpv increases the surface expression of GluR2 subunits. Bar graph showing expression of GluR2 subunits in the normal (control) , injury and injury + bpv treatment group (*p<0.05). Scale bar = 47.62 µm. B. Representative images of Western blot showing the total expression of whole GluR2 subunits in normal, injury and bpv groups. β-actin was used as a loading control. (n = 3 for each group; Compared with normal group: *p<0.05).

As expected under normal condition, a significant proportion of GluR2 clusters were localized on the membrane of neurons. The number of GluR2 cluster on cell membrane and synaptic sites was significantly reduced at 24 and 72 hours post stretch injury. But PTEN inhibitor bpv can inhibit the reduction of membrane GluR2 after injury, which had significant difference among groups (as shown in Fig. 2A). Furtherly, total expression of GluR2 protein in neurons was examined by Western blot assay. The results indicated that whether in injury or bpv treated groups, the total GluR2 protein in neurons was not altered post-injury (as shown in Fig. 2B and Figure S1).

### 3. Regulation of GluR2 subunit mediated neuroprotective effects by PTEN

**Figure 3:**
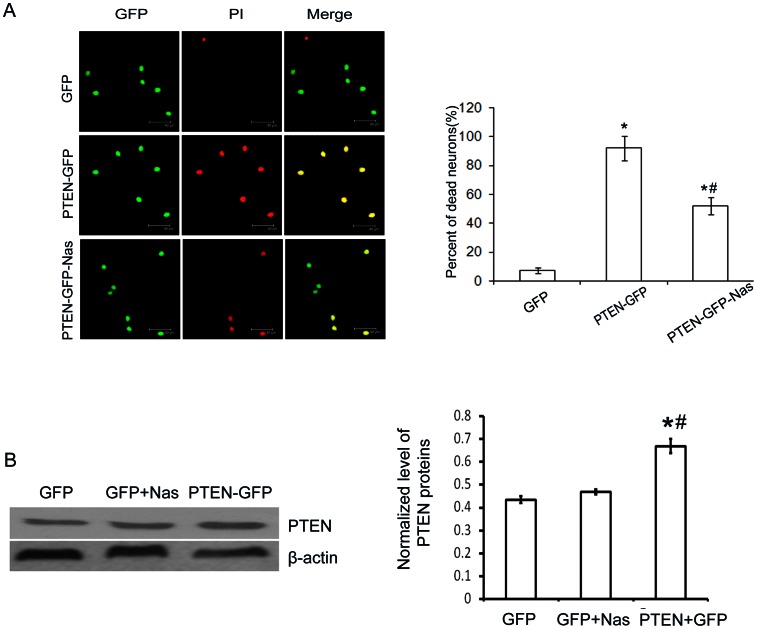
Nas reduced PTEN over-expression induced neuronal death. A. Representative images showing there were more neuronal death in neurons transfected with PTEN-GFP and Nas attenuated PTEN over-expression induced neuronal death. Bar graph showing that neuronal death was significantly increased in neurons transfected with PTEN-GFP compared with those transfected with GFP and PTEN-GFP + Nas(bar = 50.17 µm, *p<0.05 compared with GFP, ^#^ p<0.05 compared with PTEN-GFP + Nas). B. Representative images showing there were PTEN expression in normal neurons transfected with PTEN-GFP and Nas has no effect on PTEN expression in cultured neurons. Bar graph showing that the expression of PTEN in neurons transfected with PTEN-GFP was significantly increased than those in GFP and GFP+Nas groups (*p<0.05 compared with control group and #p<0.05 compared with GFP+Nas groups).

In order to indentify the regulating effect of PTEN on GluR2 subunit of AMPARs, the cultured neurons without stretch injury were transfected with PTEN over-expression vector. It was indicated that neurons transfected with PTEN**-**GFP vector realized higher expression of PTEN protein in cytoplasma of neurons (Figure S2). At the same time, the neurons transfected with PTEN-GFP appeared significantly more neuronal death than those controlled group which was transfected with GFP only. Furtherly, treatment with GluR2-lacking subunit-specific channel blockers Naspm before tranfection remarkably reduced PTEN over-expression induced neuronal death (Figure 3A), However, it was exhibited that Naspm can not affect the expression of PTEN protein in cultured neurons by Western blot examination (Fig. 3B). Therefore, it was identified that the neuroprotective effect mediated by membrane GluR2 subunit of AMPARs on neurons is regulated by PTEN.

### 4. Suppressing PTEN activity protects neurons against death caused by stretch injury

**Figure 4:**
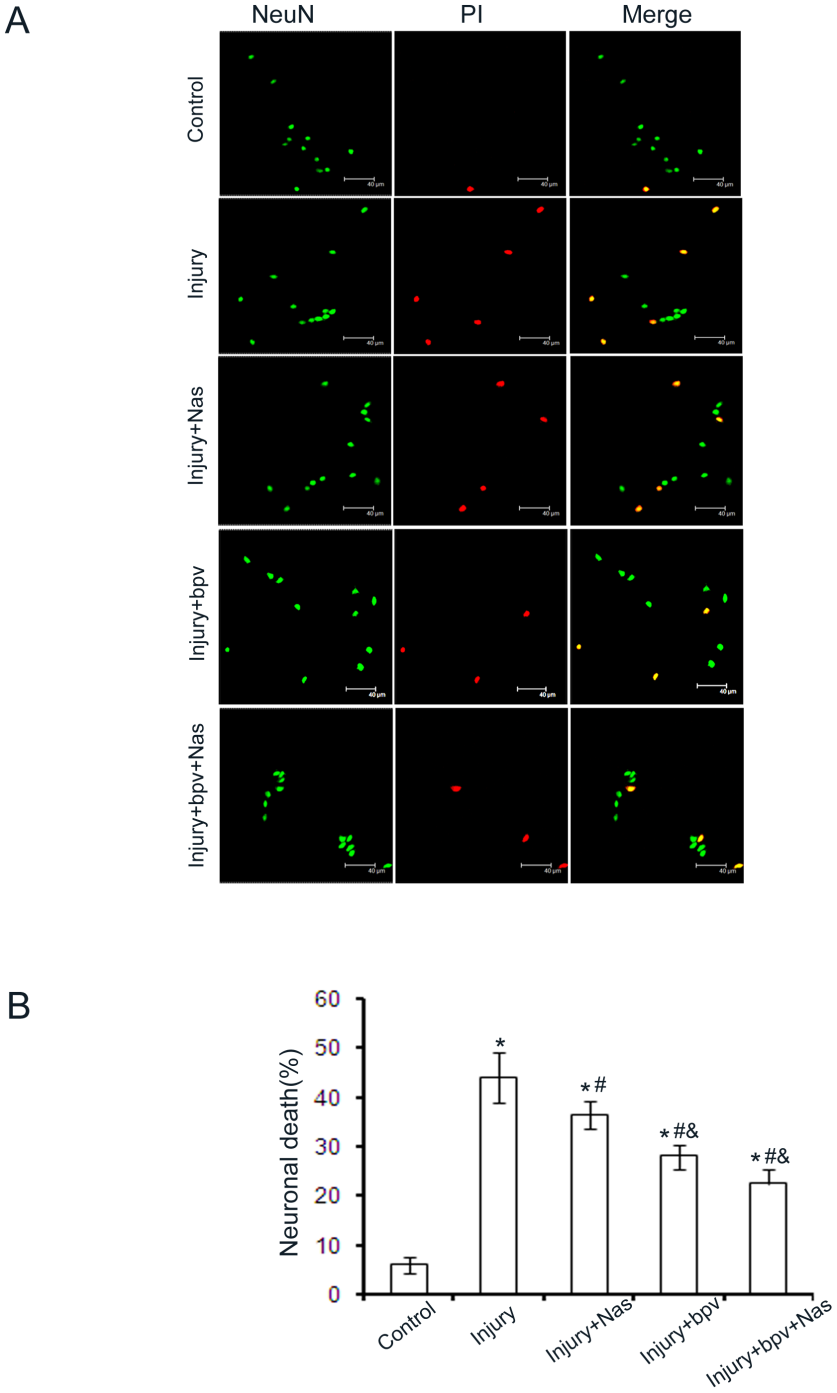
Inhibition of PTEN protects neurons from stretch-induced neuronal death. **A**. Time course of stretch-induced hippocampal neuronal death.Representative images of PI uptake and staining for the neuronal marker NeuN showing stretch-induced delayed death in hippocampal neurons at 24 h. **B**. Summary data of PI uptake. Stretch- induced neuronal death was evident at 24 h after injury (*p<0.05, difference from sham, sham + Nas, sham + bpv groups; ^#^p<0.05, difference from injury group, ^&^p<0.05 difference from injury + Nas group, ^$^p<0.05 difference from injury+bpv group. Scale bar = 75 µm.

The results of PI and NeuN double staining indicated that inhibition of PTEN by bpv remarkably reduced stretch-induced neurons death (Figure 4A), which demonstrates that down-regulating PTEN protects against neuronal death in cultured neurons. Moreover, treatment with the GluR2-lacking subunit**-**specific channel blockers Naspm significantly enhanced the bpv**-**mediated neuroprotective effect (Figure 4B).

## Discussion

TBI and transient global ischemia usually cause delayed neuronal death and deficits of cognitive and motor functions. However, the underlying mechanisms remain unclear. Ca^2+^-permeable AMPA receptors have a crucial role not only in synaptic plasticity, but also in the excitoxicity associated with several neural injury [3], [5]. Although Ca^2+^-permeable AMPA receptors have been reported to take part in the process of ischemic brain injury [12], [20], [21], its exact role in traumatic neural injury has not been well explained. In this experiment it was indicated that cultured neurons of hippocampus are highly vulnerable to mild trauma injury. and the expression of GluR2 subunit of AMPARs on the surface of neurons reduces dramatically post-injury, but the total GluR2 expression in neurons was not changed. Furthermore, inhibiting GluR2-lacking AMPARs with Naspm significantly prevented those injured neurons from death. As it was known that a striking feature in TBI is an early rise in intracellular Ca^2+^ [11], [22], [23]. It was recently reported that that GluR2-lacking AMPARs trafficking mediated post-traumatic calcium overload, and initiated ultimately progressive cell death [5], [9].

Although considerable evidences indicated that GluR2-lacking AMPARs trafficking are usually associated with several neurological disorders and diseases, the further mechanisms about the reduction of GluR2 subunit on the neuronal membrane following injury has yet to be determined. In this experiments, it was observed that the expression of PTEN protein and mRNA in cultured neurons both increased significantly post-injury, which was contrary to the expression of GluR2 subunit on the membrane surface of neurons. At the same time, inhibition of PTEN activity by its specific blocker bpv reduced the death of hippocampal neurons after stretch injury. These results indicated that increased PTEN in neurons post-injury may be related to the decreasing expression of GluR2 subunit on the membrane surface of neurons.

Modulation of AMPARs by kinases and phosphatases is a crucial cellular process that regulates diverse neuronal functions [24], [25]. However, little is known about the role of membrane AMPARs in this regulatory process. it was demonstrated in normal conditions that high expression of PTEN through transfection may lead to the death of neurons. On the other hand, specific inhibition of the activity of PTEN by bpv resulted in the increasing expression of GluR2 subunit on neuron surface after stretch injury which protected neurons against traumatic death. Interestingly, our data furtherly exhibited that treatment with both GluR2-lacking AMPARs inhibitor Naspm and PTEN specifical inhibitor bpv had similar protective effect to that of bpv on neurons subjected to stretch injury. Further more, it was identified that Naspm had no effect on the expression of PTEN by western blot examination. This results indicated that the protein phosphatase activity of PTEN acted as a crucial upstream signal to regulate the function of AMPAs subunit GluR2 trafficking.

Taken together, in this work, it was demonstrated that increased PTEN expression induced reduction of Ca^2+^-impermeable GluR2 subunit of AMPARs on the surface of injured hippocampal neurons, which at last led to death of neurons.Suppressing PTEN activity via its blocker bpv inhibits GluR2-lacking AMPARs and protects against hippocampal neuronal from death after injury. Therefore, this study identified an alternative mechanism underlying TBI by which the regulation of GluR2 subunit of AMPARs by PTEN mediates the delayed hippocampal neuronal death following injury.

## Supporting Information

Figure S1 **The immunocytochemical staining of total GluR2 in cultured neurons through penetrating the cells with Triton-100.**
(TIF)

Figure S2 **The immunocytochemical staining of PTEN in neurons before and post-transfection.**
(TIF)
