# Supplementary figures and images for: Inhibiting PTEN Protects Hippocampal Neurons against Stretch Injury by Decreasing Membrane Translocation of AMPA Receptor GluR2 Subunit
Source: PLoS One. 2013 Jun 17;8(6):e65431. doi: 10.1371/journal.pone.0065431 (PMC3684616; doi:10.1371/journal.pone.0065431)

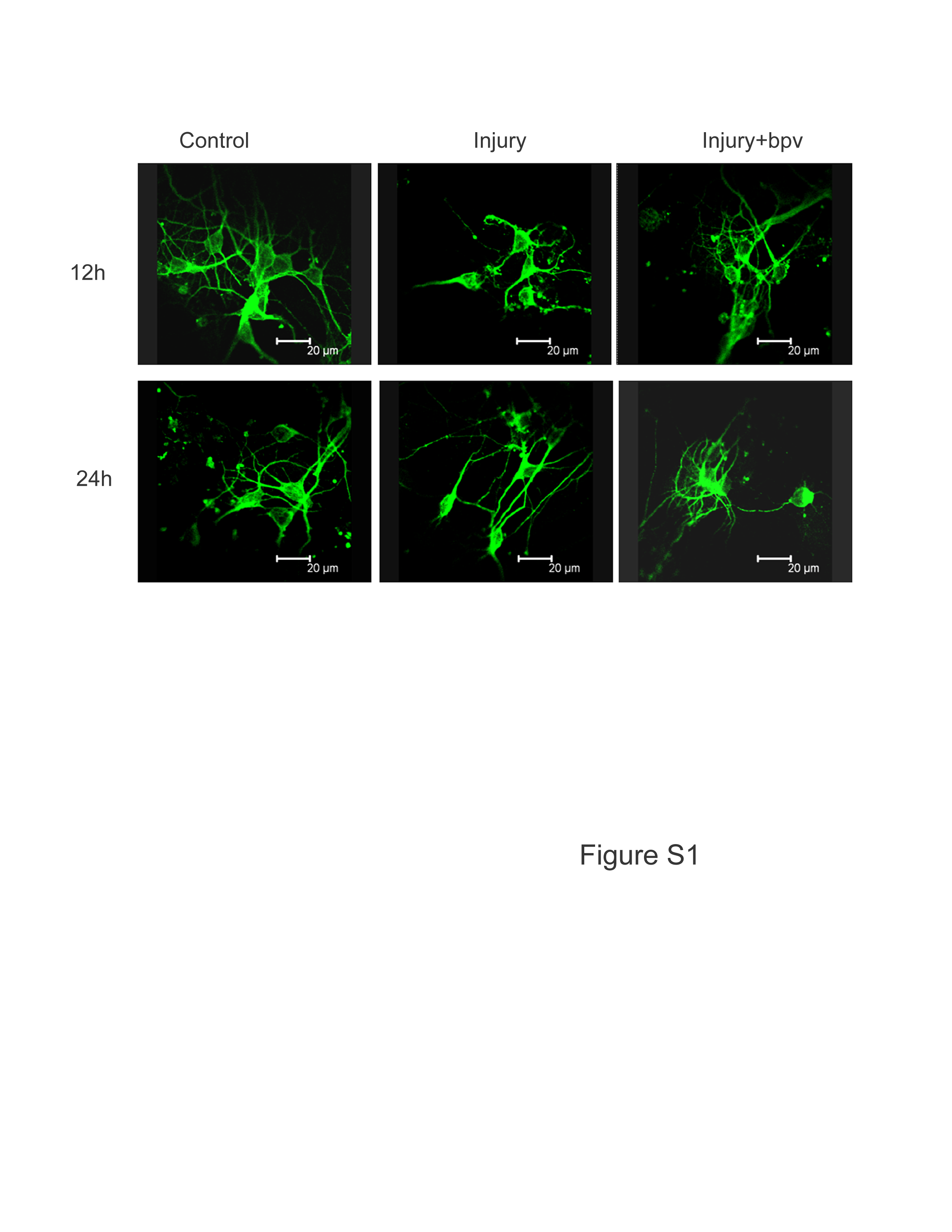

Supplement: Figure S1 — The immunocytochemical staining of total GluR2 in cultured neurons through penetrating the cells with Triton-100. (TIF) [file pone.0065431.s001.tif]

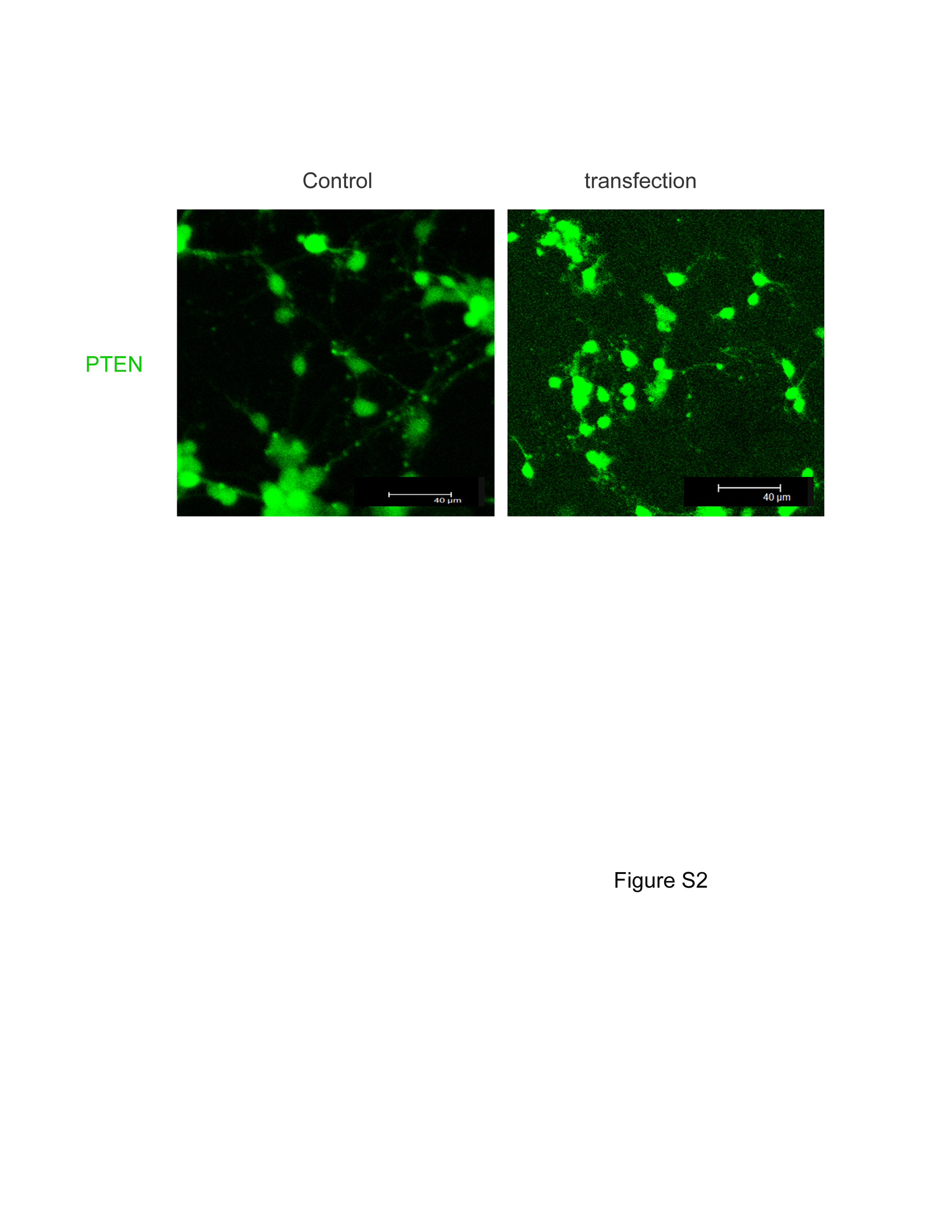

Supplement: Figure S2 — The immunocytochemical staining of PTEN in neurons before and post-transfection. (TIF) [file pone.0065431.s002.tif]
